# Towards a better understanding of human iNKT cell subpopulations for improved clinical outcomes

**DOI:** 10.3389/fimmu.2023.1176724

**Published:** 2023-04-19

**Authors:** Alex Look, Daniel Burns, Ivo Tews, Ali Roghanian, Salah Mansour

**Affiliations:** ^1^ NIHR Biomedical Research Centre, School of Clinical and Experimental Sciences, Faculty of Medicine, University of Southampton, Southampton, United Kingdom; ^2^ Biological Sciences, University of Southampton, Southampton, United Kingdom; ^3^ Institute for Life Sciences, University of Southampton, Southampton, United Kingdom; ^4^ Antibody and Vaccine Group, Centre for Cancer Immunology, School of Cancer Sciences, Faculty of Medicine, University of Southampton, Southampton, United Kingdom

**Keywords:** iNKT cell, CD1d, cancer, lipid, immunotharapy

## Abstract

Invariant natural killer T (iNKT) cells are a unique T lymphocyte population expressing semi-invariant T cell receptors (TCRs) that recognise lipid antigens presented by CD1d. iNKT cells exhibit potent anti-tumour activity through direct killing mechanisms and indirectly through triggering the activation of other anti-tumour immune cells. Because of their ability to induce potent anti-tumour responses, particularly when activated by the strong iNKT agonist αGalCer, they have been the subject of intense research to harness iNKT cell-targeted immunotherapies for cancer treatment. However, despite potent anti-tumour efficacy in pre-clinical models, the translation of iNKT cell immunotherapy into human cancer patients has been less successful. This review provides an overview of iNKT cell biology and why they are of interest within the context of cancer immunology. We focus on the iNKT anti-tumour response, the seminal studies that first reported iNKT cytotoxicity, their anti-tumour mechanisms, and the various described subsets within the iNKT cell repertoire. Finally, we discuss several barriers to the successful utilisation of iNKT cells in human cancer immunotherapy, what is required for a better understanding of human iNKT cells, and the future perspectives facilitating their exploitation for improved clinical outcomes.

## Introduction

Recent therapeutic advances utilising immune-checkpoint inhibitors (ICI) have revolutionised the cancer immunotherapy field which was mainly facilitated by the introduction of monoclonal antibodies (mAb) targeting the T cell immune inhibitory molecules PD-1 and CTLA-4. Antibodies targeting these molecules have been used as single agents or in combination for the treatment of many cancers including previously untreatable solid tumours, such as end-stage melanoma (1). However, despite the wide success of ICI, these therapies are only efficacious in a subset of cancer patients (2). Therefore, novel and more effective complementary cancer immunotherapies are needed.

Several cellular therapeutic strategies have taken centre stage, including adoptive T cell and chimeric antigen receptor (CAR) T cell therapy (3). One particular population of T cells, namely the invariant natural killer T cells (iNKT) are the focus of intense research as they can deliver powerful anti-tumour responses (4, 5). iNKT cells are not restricted to polymorphic human leukocyte antigen (HLA) and bind exclusively to the monomorphic CD1d molecule and therefore do not cause graft versus host disease (6–8). Thus, there is an untapped potential for iNKT cells to serve as an off-the-shelf therapy (9–11). To realise the potential of iNKT cells, we must develop a better understanding of human iNKT cell biology to improve subsequent iNKT cell immunotherapies.

## iNKT cells

iNKT cells are an unconventional subset of αβ T cells that specifically recognise lipids and glycolipids presented by the monomorphic MHC-like molecule CD1d (12–14). As they leave the thymus into the periphery as fully primed and matured T cells, they are considered an ‘innate-like’ subset that can bridge the gap between innate and adaptive immunity (15). They are also described as attractive targets for cancer immunotherapy as they play important roles in tumour immunosurveillance and anti-tumour immunity (5, 11, 16, 17).

iNKT cells are T lymphocytes that display properties of both T cells and NK cells, as defined by specific cell surface markers such as NK1.1 and NKG2D (10, 18–20). Human iNKT cells express a semi-invariant TCR, with the α chain consisting of an invariant Vα24-Jα18 chain paired with a Vβ11 chain (21). In contrast, the chain pairing differs in mice where an invariant Vα14-Jα18 chain pairs with one of three β chains (Vβ8, Vβ7, or Vβ2) (22).

iNKT cells, similarly to conventional αβ T cells, begin their journey in the thymus where they undergo a rigorous selection process and subsequently migrate to peripheral tissues in a matured state able to perform effector functions without priming (15, 23). Human iNKT cells can be identified using mAbs (TCR Vα24 Jα18 clone 6B11), by reactivity to the prototypical synthetic iNKT agonist glycolipid α-galactosylceramide (αGalCer) and by binding to CD1d-αGalCer tetramers (24). Human iNKT cells can be divided into CD4^-^CD8^-^ double negative (DN), CD4^+^, CD8^+^ or CD4^+^CD8^+^ double positive (DP) populations (25, 26). In contrast to humans, murine iNKT cells are more frequent and only occur as either CD4^+^ or DN T cells (27, 28).

In humans, iNKT cells are predominantly located within the thymus, liver, bone marrow, spleen, and peripheral blood (for more information about the distribution of iNKT cells in mouse and human tissues, we refer the reader to an excellent review by Crosby and Kronenberg (27)). Within human peripheral blood, iNKT cells make up 0.01%-0.2% of T cells with no differences between gender; however, they can range from undetectable to over 1% (25, 26). Even when iNKT cells are in their resting state they carry pre-formed mRNA enabling production of T helper 1 (Th1) and T helper 2 (Th2)-type cytokines (29). Upon recognition of CD1d, iNKT cells rapidly secrete copious immunomodulatory cytokines such as interferon gamma (IFN-γ), tumour necrosis factor alpha (TNF-α), interleukin (IL)-4, and IL-17 to instigate and influence downstream immune responses (29, 30). However, iNKT cells have also been shown to secrete IL-2, IL-5, IL-6, IL-10, IL-13, IL-21, TGF-β and GM-CSF, as well as several chemokines (31).

Activation of iNKT cells results in TCR downregulation, proliferation, and prolonged cytokine secretion (32, 33). The repertoire of Th1 and Th2 type cytokines produced by iNKT cells is modulated by the strength of the iNKT cell TCR signalling, as well as by the iNKT agonist and the type of antigen-presenting cells (APCs) presenting iNKT antigens (11, 15). Consequently, iNKT cells serve as a source of cytokines that activate and recruit other cell types including APCs early during immune responses, while activated APCs subsequently direct the ensuing adaptive immune response which help protect from infection and tumour growth (15, 34).

In addition to activation via iNKT cell TCR recognition of CD1d-lipid complexes, iNKT cells may also be activated by cytokines, such as IL-12 and IL-18 (27, 35). iNKT cells are some of the first responders during an immune response. Indeed, activated iNKT cells upregulate their IL-12 receptor and CD40L; through cross talk they induce the maturation of dendritic cells (DCs), and the subsequent production of IL-12 by the DCs (36). IL-12 secretion by DCs increases IFN-γ production by iNKT cells, leading to a positive feedback loop for Th1 immunity (10, 36). The maturation of DCs leads to increased production of IFN-γ by NK cells along with upregulation of MHC class I and II antigen presentation to T cells (37). This feature of iNKT cell biology to “jump-start” the ensuing innate and downstream adaptive immune response is central to exploiting iNKT cells in immunotherapy to promote anti-cancer immunity (10, 15, 37).

While the CD1d-iNKT interaction is relatively well studied in the mouse, the human iNKT system is less well understood. There have been several clinical trial efforts attempting to use the strong iNKT agonist lipid αGalCer to activate this subset of T cells or through using adoptive iNKT cell immunotherapy (**Table 1**). However, plenty of evidence suggests the existence of a more diverse human iNKT repertoire, leading to investigations of specific subsets which has the potential to improve future iNKT-targeted cell therapies (38).

**Table 1:** A summary of first-in-man clinical trials with iNKT-based immunotherapies.

Treatment Type	Phase	Number of Patients Completing trial/Enrolled	Tumour Type	Clinical Outcomes (Number of patients)	Immune Response	Reference
αGalCer (i.v.)	I	24	Solid Tumours	SD	Increase in serum cytokine (TNF-α)	Giaccone et al. 2002 (20)
αGalCer-pulsed immature MoDC (i.v.)	I	12	Solid tumours with metastatic malignancy	Reduction of serum tumour markers (2) Necrosis of tumour (1)	Increase in serum IFN-γ IL-12 increased in 6 donors	Nieda et al. 2004 (21)
αGalCer-pulsed mature DC (i.v.)		5/6	Solid tumours and myeloma	Reduction of M protein (3) SD (1)	>100 fold expansion iNKT cells Serum increase in IL-12 and IFN-γ	Chang et al. 2005 (22)
αGalCer-immature DCs- rich APCs (i.v.)	I	9/11	Lung cancer	SD (5)	Increase in iNKT cells (1). No patients meet criteria PR or CR	Ishikawa et al. 2005 (23)
αGalCer-activated iNKT (i.v.)	I	6	Lung cancer	SD (4), PR (2)	Increase in iNKT cells, increase in IFN-γ producing cells	Motohashi et al. 2006 (24)
αGalCer-pulsed antigen presenting cells (nasal)	I	9	unresectable or recurrent HNSCC	PR (1), SD (5), PD (3)	Increase in iNKT (4) Significant increase in IFN-γ producing cells (8/9)	Uchida et al. 2008 (25)
αGalCer-APCs (via nasal submucosa) αGalCer-activated iNKT (intra-arterial infusion)		8	HNSCC	SD (4), PR (3), PD (1)	Increase in iNKT cells, increase in IFN-γ producing cells	Kunii et al. 2009 (26)
αGalCer-pulsed APCs (i.v.)	I-II	17/23	Advanced and recurrent NSCLC	SD (5), PD (12)	Increased number of IFN-γ producing cells in the peripheral blood (10). Median survival time higher in responders	Motohashi et al. 2009 (27)
αGalCer-APCs (via nasal and oral submucosa)		17	HNSCC	No anti-tumour activities detected	Increase of iNKT and IFN-γ producing cells	Kurosaki et al. 2011 (28)
αGalCer-immature DCs (i.v. and i.d.)	I	12	Solid tumours	SD (3) PR (3)	Increase in serum IFN-γ. Significant iNKT cell increase	Nicol et al. 2011 (29)
αGalCer-APCs (via nasal submucosa) αGalCer-activated iNKT (intra-arterial infusion)	II	10	HNSCC	SD (5), PR (5)	Tumour regression (5), increase in iNKT in cancerous tissue which was associated with tumour regression (7)	Yamasaki et al. 2011 (30)
αGalCer-APCs (i.v.)		4	Lung cancer	–	Infiltration and activation of iNKT	Nagato et al. 2012 (31)
αGalCer-mature DCs + lenalidomide (i.v.)		6	Asymptomatic myeloma	Led to reduction in tumour-associated monoclonal immunoglobulin in 3 of 4 patients with measurable disease	Increase of iNKT, NK, monocytes, eosinophils	Richter et al. 2013 (32)
Expanded iNKT transfer (i.v.)	I	9	Stage IIIB–IV melanoma	SD (3), further treatment (3), dead (3)	Increase in iNKT cells and IFN-γ production	Exley et al. 2017 (33)
Trans-bronchial injection αGalCer - APCs	I	21	Advanced or recurrent NSCLC	PR (1), SD (8)	Increased iNKT cell numbers were observed in PBMCs from eight cases, and IFN-γ producing cells were increased in the peripheral blood of 10 cases	Ishibashi et al. 2020 (34)
iNKT cells (i.v.)	I-II	120	Advanced solid tumour			NCT02562963
iNKT cells (i.v.) combined with transcatheter arterial chemoembolization	II-III	144	Advance HCC			NCT04011033
agenT-797 infusion	I	20	Relapsed/Refractory Multiple Myeloma			NCT04754100
agenT-797 infusion	I	30	Solid tumours			NCT05108623
Cyclophosphamide and fludarabine will be administered prior to the GINAKIT cells (GD2-CAR iNKT cells).	I	36	Neuroblastoma			NCT03294954
Allogenic CD19-CAR iNKT cells	I	48	B cell Malignancies			NCT03774654
Autologous CD19-CAR iNKT	I	20	Acute Lymphoblastic Leukemia, B-cell Lymphoma, Chronic Lymphocytic Leukemia			NCT04814004
Administration of PRECIOUS-01, an iNKT cell activator threitolcermaide-6 and NY-ESO-1 encapsulated in a nanoparticle	I	15	Advanced solid tumour			NCT04751786
Infusion of iNKT cells and CD8+T cells	I-II	40	Non-small Cell Lung Cancer, Small Cell Lung Cancer, pancreatic cancer, Hepatocellular Carcinoma, Gastric Cancer, Renal Cell Carcinoma			NCT03093688
Autologous iNKT Cells + Tegafur +Interleukin-2	I	18	HCC			NCT03175679
GM-CSF + iNKT	I	9	Malignant Melanoma			NCT00631072

APC, antigen-presenting cell; SD, stable disease; PR, partial response; CR, complete response; PD, progressive disease; HCC, hepatocellular carcinoma; HNSCC, head and neck squamous cell carcinoma; NSCLC, non-small cell lung cancer; i.d., intradermal injection; i.v., intravenous injection.

## The anti-tumour mechanisms of iNKT cells

**Figure 1:**
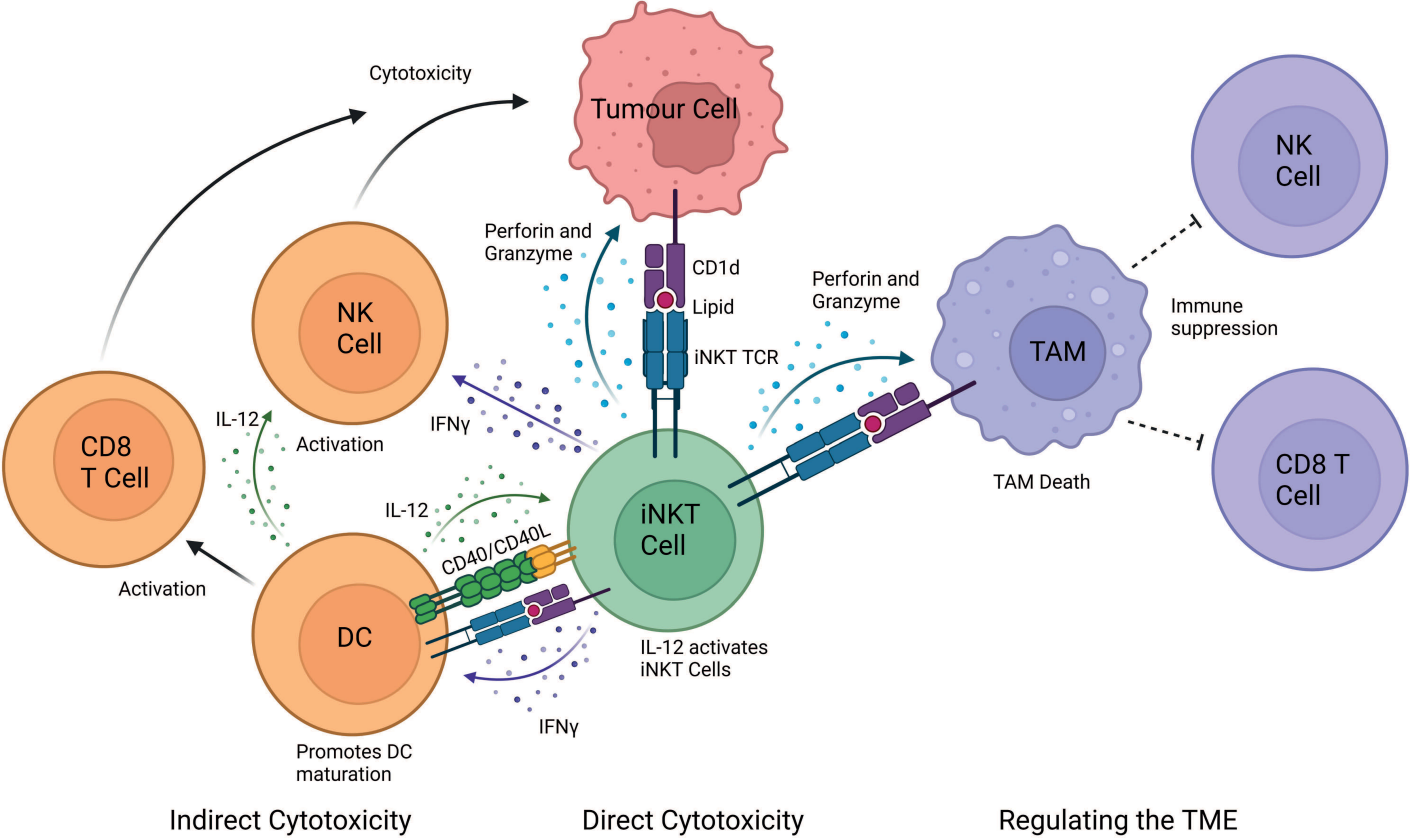
iNKT cell mediated mechanisms of tumour cytotoxicity. iNKT can exert their cytotoxic function either directly, indirectly and by regulating the tumour microenvironment (TME). In the direct mechanism, iNKT cells can recognise the tumour through the engagement of their iNKT cell TCR with a CD1d-lipid complex expressed on the surface of the tumour cell. iNKT cells can indirectly exert cytotoxic functions by interacting with other immune cells in the TME. IFN-γ released by iNKT cells can activate NK cells to perform their anti-tumour cell effector functions. Additionally, IFN-γ release activates the maturation of DCs and stimulates CD8^+^ T cells. iNKT cells and DCs reciprocally activate each other in a CD40-CD40L and CD1d-lipid/TCR antigen-dependent manner, which further stimulates iNKT cells. iNKT cells also regulate the TME by engaging with CD1d-lipid complexes expressed on tumour-associated macrophages (TAMs). This interaction promotes iNKT killing of immunosuppressive TAMs, consequently providing a less immunosuppressive environment where tumour-infiltrating NK cells and CD8^+^ cytotoxic T cells can better perform their functions. Image created in BioRender.

Mouse studies have demonstrated that iNKT cells exert powerful anti-tumour responses, however, translation into humans has proved difficult (11, 39). It is now unanimously accepted that iNKT cells play a pivotal role in anti-tumour immunity both in mice and humans (11). Primarily there are three mechanisms of action through which iNKT cells elicit a cytotoxic response: (i) direct tumour lysis, (ii) recruitment and activation of other innate and adaptive immune cells by initiating a Th1 cytokine cascade, and (iii) regulation of immunosuppressive cells in the tumour microenvironment (TME) (**Figure 1**).

### Direct tumour lysis

Direct iNKT cytotoxicity occurs mainly against cells expressing cell surface markers which are recognised by iNKT cell surface receptors. Some tumour cells highly express CD1d molecules, for example tumours of myelomonocytic and B cell lineage origin as well as some solid tumours, such as glioblastoma (34, 40). The CD1d molecules on tumour cells bind and present endogenous tumour-associated glycolipids. Through TCR-mediated recognition of the tumour CD1d-lipid complex, iNKT cells can directly promote lysis of the CD1d^+^ tumour cells (41). Surface expression of CD1d on tumour cells is assumed to directly correlate with iNKT cell-mediated cytotoxicity, although this seems to depend on the target cell in question (42). Nevertheless, in certain cases higher expression of CD1d can result in increased tumour cell lysis, whereas lack of CD1d expression in tumours leads to their escape from recognition promoting tumour progression (34, 43–46).

iNKT cells exert their direct cytotoxic functions either through the death receptor mediated pathway also known as the extrinsic apoptotic pathway, or the cytotoxic granule release mechanism. Both require cell-to-cell interaction and require activation of executioner caspases (47). The death receptor pathway requires binding of ligand expressed on the iNKT cell with its receptor on the target cell (e.g., Fas ligand [FasL]/receptor [FasR]). In contrast, the granule exocytosis pathway requires the pore forming protein perforin, and a family of serine proteases known as granzymes to cleave and activate effector molecules within the target cell (48).

It has been shown that cytotoxicity towards Jurkat cells was CD1d independent and relied mostly on FasL/FasR interactions (45). Additionally, in human T cell lymphoma patients, CD1d levels were elevated and defects in the frequency and function of circulating iNKT cells were observed (45). However, the presence of CD1d on tumour cells is not a requirement, as iNKT cells can recognise leukaemia cells in a CD1d-independent manner and degranulate releasing Th1 cytokines towards the CD1d^-^ leukaemia. This response is enhanced by NK cell activating receptors, such as, NKG2D, 2B4 (CD244), DNAM-1, LFA-1 and CD2 which are also expressed on the iNKT cells (19, 47). Therefore, these studies suggest that the iNKT cell mediated anti-tumour responses may critically depend on the inherent quality of the iNKT cell population and the type of tumour cells encountered.

Perforin and granzymes are the major constituents of cytotoxic granules which are stored in the cytosol of iNKT cells or produced upon activation (48, 49). Upon iNKT cell engagement with a target cell, the granules polarise towards the immunological synapse releasing granzymes and perforin via exocytosis. Perforin embeds in the target cell membrane to allow transfer of granzymes into the cytosol of the target cell (50, 51). In humans, five granzymes named A, B, H, K, and M have been identified. Granzyme B is the most potent cell death inducing molecule of the group and can induce cell death even at low concentrations (48). This is due to its ability to provoke cell death in either a caspase-dependent manner through pro-caspase 3, 7 and 8 cleavage or in a caspase independent manner (47, 48). Effector caspase signalling initiates the release of a DNase involved in DNA damage from its inhibitor, thereby inducing target cell death (52).

Another cytotoxic molecule within the granules of human iNKT cells is granulysin (52), a member of the saposin-like family of proteins (52, 53). Granulysin is a 15 kDa molecule which is cleaved at both the amino and carboxyl termini to yield a 9 kDa isoform that has cytotoxic properties and is stored in cytotoxic granules along with perforin and granzymes. Granulysin has a wide spectrum of activity and is involved in the immune response to several pathogens including, fungi, parasites, bacteria, and protozoa. The presence of granulysin has been correlated with reduced cancer progression (54). Elevated granulysin concentrations have been detected in gastric carcinoma patients with less severe disease than those with advanced stage gastric carcinomas (54, 55). The cytotoxic ability of the 9 kDa isoform of granulysin is thought to be responsible for the killing of tumour cells by altering the membrane permeability of the cell, which leads to an increase in intracellular calcium, thus, inducing tumour cell lysis (52, 56).

### Recruitment of innate or adaptive immune cells

In the second cytotoxic response mechanism, iNKT cells can activate and recruit innate and adaptive immune cells, such as DCs, NK, B and T cells upon engagement of the iNKT cell TCR (57). Upon activation, iNKT cells secrete Th1 and Th2 cytokines which leads to reciprocal activation of effector lymphocytes (11). For example, IFN-γ release activates the maturation of DCs and stimulates CD8^+^ T cells. iNKT cells and DCs reciprocally activate each other in a CD40-CD40L and CD1d-lipid/TCR antigen dependent manner, thus initiating adaptive anti-tumour immunity (58). iNKT cells enhance tumour immunity by subduing the actions of tumour supporting cells, such as tumour-associated macrophages (TAMs) and myeloid-derived suppressor cells (MDSC) (11). Additionally, cytokine release, such as IL-2, IL-12 and IFN-γ by iNKT cells leads to the activation and expansion of NK cells into lymphokine-activated killer (LAK) cells. These activated LAK cells upregulate effector and adhesion molecules, such as perforin, NKp44, granzymes, FasL and TRAIL and secrete IFN-γ to adhere to and lyse tumour cells (11, 59).

### Regulating the tumour microenvironment

In established tumours, TAMs are typically immunosuppressive cells which reside within the TME and supress immune cell function (60–62). TAMs contribute to the tumour progression by enhancing angiogenesis, enhancing tumour cell invasion, and suppressing NK and T cell responses (63, 64). iNKT cells have been demonstrated to co-localise with CD1d-expressing TAMs in neuroblastoma and kill TAMs in a CD1d-restricted manner (65). iNKT cells can indirectly mediate anti-tumour activity via the removal of TAMs, thereby modulating the favourable environment of tumour cells, by removing their immunosuppressive function (34). In addition, iNKT cells interfere with the effects of CD1d MDSC-mediated immune suppression. MDSCs can accumulate during tumour growth, aiding tumour immune escape and progression (66). However, iNKT cells can prevent the suppressive activity of MDSCs in a CD1d- and CD40-dependent manner (67).

## Preclinical studies: iNKT cells in cancer

Seminal studies in the 1990s demonstrated that αGalCer was a potent activating ligand of mouse and human iNKT cells (13). αGalCer is presented by CD1d expressing APCs to selectively stimulate iNKT cells. However, iNKT cell activation requires costimulatory signals provided by CD40/CD40L and B7/CD28 interactions (13). Several studies have demonstrated that αGalCer exhibits anti-tumour properties against a variety of tumours, including B16 melanoma (68) and 3LL lung carcinoma (69). Therapeutic administration of αGalCer markedly reduces the number of B16 lung metastases in wild type (WT) C57BL/6 mice. This anti-tumour activity was completely abolished in Jα18^−/−^ (deficient in iNKT cells), CD1d^−/−^ (deficient in both NKT cell subsets) and RAG-1^−/−^ (lack mature B and T lymphocytes) mice, strongly suggesting that iNKT cells were responsible for the anti-tumour effects (20). Additionally, αGalCer treatment following chemotherapy (cisplatin) delayed tumour cell proliferation and increased tumour cell death in mesothelioma AB12 laden mice (70). In mice with liver metastases of adenocarcinoma Colon26 cells, administration of the αGalCer synthetic analogue KRN7000, one day after tumour inoculation significantly inhibited tumour growth in the liver with a potency similar to that of IL-12 (71). Even when treatment was given after nodule formation (day 3), tumour regression was observed (71). Anti-tumour activity of KRN7000 in mice with spontaneous liver metastases of reticulum cell sarcoma M5076 tumour cells suppressed the growth of established liver metastases and resulted in the prolongation of survival time (72). An increase in iNKT cell numbers and IL-12 production by hepatic lymphocytes was markedly enhanced in KRN7000-treated mice (72). Together, these results suggest that the *in vivo* anti-tumour effects of KRN7000 are dependent on iNKT cells and endogenous IL-12 production.

Adoptive transfer of αGalCer-loaded APCs was explored as an alternative approach to stimulate iNKT cells. DCs pulsed with αGalCer can effectively induce potent anti-tumour cytotoxicity by their specific activation of Vα14^+^ iNKT cells, resulting in the inhibition of tumour metastasis *in vivo* (73). Moreover, a complete inhibition of B16 melanoma metastasis in the liver was observed when αGalCer-pulsed DCs were injected, even seven days after transfer of tumour cells to syngeneic mice, when small but multiple metastatic nodules were already formed (73).

Mice with a deletion of the *Trαj18* gene segment do not express the Vα14 *Trαj18* TCR and were found to exclusively lack iNKT cells; while they maintained numbers of lymphocytes that were almost identical to WT mice (5). The resulting iNKT deficient mice were no longer able to mediate IL-12-induced rejection of B16 melanoma tumours (5). Adoptive transfer of IL-12-activated Vα14 iNKT cells prevented hepatic metastasis of B16 melanoma in mice (74). This suggested the involvement of direct cytotoxic mechanisms by iNKT cells rather than cytokine-mediated immune responses. Furthermore, adoptive transfer of iNKT cells into iNKT cell-deficient (Jα18^-/-^) mice restored tumour surveillance and protected against methylcholanthrene-induced fibrosarcoma in the absence of exogenous stimulatory factors (4). However, the production of IFN-γ by iNKT and other lymphocytes was essential for protection. Using humanised NSG mice, a recent study showed that tumour localised administration of αGalCer can significantly enhance iNKT cell-mediated anti-tumour capacity against solid tumours (75).

The studies described above utilised the Jα18-deficient mice which were described in 1997 (5). Recently, a study revealed that about 60% of TCRα repertoire diversity was lacking in these mice due to the absence of *Trαj* gene segments upstream of *Trαj18* (76). Subsequently this generated concern regarding the validity of the experimental conclusions, such as those of Toura et al., 1999 (73). The Jα18-deficient mouse strain was again called into question when a report showed that Jα18^−/−^ mice are also defective in mucosal-associated invariant T (MAIT) cells in both the thymus and peripheral organs (77). Therefore, caution is advised when interpreting data from the TCRJα18^−/−^ mouse strain. Consequently, the group that described the original Jα18^−/−^ mice generated a novel *Trαj18*-deficient mouse line by specifically targeting the *Trαj18* gene segment. Apart from the absence of *Trαj18*, these mice had an undisturbed TCRα repertoire. Next generation sequencing detected normal generation of Vα19Jα33 expressing MAITs, whose development was abrogated in the originally described Jα18^−/−^ mice (78). Using a B16 melanoma liver metastasis model with mice bearing metastatic melanoma nodules in the liver, intravenous administration of αGalCer-pulsed DCs as described previously resulted in the complete eradication of melanoma metastasis in WT but not in Trαj18^-^/^-^ mice (78). Indeed, the tumour growth in the DC-αGalCer treated *Trαj18^-/-^* mice was similar to that in the vehicle-treated WT and *Trαj18^-/-^* mouse groups, demonstrating the absolute requirement for activated iNKT cells in tumour rejection (78).

Taken together, there is now conclusive evidence from preclinical studies to suggests that iNKT cells exhibit powerful anti-tumour activity and are involved in cancer immunosurveillance (10, 16). They likely act as early warning systems to initiate an anti-tumour response which is subsequently performed by dedicated effectors such as NK cells and/or cytotoxic T lymphocytes (15). The potent iNKT cell-derived IFN-γ production and early activation of effector cells such as NK and CD8^+^ T cells suggests that iNKT cells can be rapidly stimulated by glycolipids on the tumour cell or by other stimuli. Effector cells then directly attack the tumour through direct perforin-dependent lysis and indirectly through raising an IFN-γ response (15). Early recognition of the tumour appears to be key, as delayed transfer of iNKT cells provides less protection (4).

## Translation of iNKT immunotherapy into humans

Following promising results in preclinical models, many human clinical trials have begun to exploit iNKT cells to harness their anti-tumour potential. Initially, cancer patients with solid tumours were intravenously injected with soluble αGalCer. However, while αGalCer treatment was well tolerated, it failed to initiate an effective clinical response (79). As preclinical studies suggested that DCs loaded with αGalCer confers better immune responses *in vivo* (73), αGalCer-pulsed DCs were trialled in patients with advanced and recurrent non-small cell lung cancer, head and neck squamous cell carcinoma and myeloma (80–84). Different strategies were exploited to improve this treatment, which included utilising different types of APC or alternative routes of administration. Again, despite good tolerance and an increase in iNKT cell numbers, clinical benefits were limited (80, 81, 83–85).

Since adoptive T cell therapy for cancer treatment has long been established, several groups begun exploiting expanded iNKT cell products. Adoptive transfer of iNKT cells into patients with melanoma, head and neck squamous cell carcinoma, lung cancer and other solid tumours revealed increased iNKT numbers *in vivo* and increased levels of IFN-γ (86–88). While adoptive transfer was well tolerated, further improvements would be required to achieve a significant clinical response in patients (11).

Studies that exploited iNKT cells in CAR-T cell therapy showed promising results in murine preclinical studies (6, 89). CAR-iNKT cells exhibited significantly better *in vivo* responses than traditional CAR-T cells in mice when targeting GD2^+^ neuroblastomas and CD19^+^ lymphomas (6, 89). CAR-iNKT induced little graft versus host disease and their efficacy was augmented through their dual targeting ability of CD1d and CD19/GD2. Indeed, CAR-iNKT targeted both GD2^+^ neuroblastoma cells and CD1d^+^ TAMs. Additionally, CD1d^+^ CD19^+^ lymphoma cells have been targeted by CAR-iNKT cells in a dual pronged attack which effectively localised to the tumour site, had potent anti-tumour activity, and significantly improved the long-term survival of treated mice (89). While these studies indicated that iNKT cells are a highly efficient platform for CAR-based immunotherapy in mice, clinical trials are now ongoing for patients with B cell lymphoma, leukaemia and glioblastoma, and promising initial results were reported in humans (90). Therefore, these translational studies open a promising avenue for iNKT targeting cancer therapies in patients, but they lack clinical efficacy at present (91). iNKT immunotherapy will require further improvements to achieve effective clinical outcomes.

## The future of iNKT cancer immunotherapy in humans

A key challenge for iNKT cell cancer immunotherapy in humans is a defective iNKT cell repertoire in human cancer patients both quantitatively and qualitatively. Several studies have demonstrated reduced iNKT cell numbers in the peripheral blood of cancer patients (39, 79, 92). Furthermore, iNKT cells derived from cancer patients release reduced levels of IFN-γ as they tend to exhibit a Th2 phenotype and CD1d expression can be downregulated in tumours, which abrogates the efficacy of the direct iNKT cell-mediated immune response (46). Several mechanisms have been postulated that may explain the suboptimal efficacies of iNKT cell anti-tumour response in clinical trials (9, 11), such as the induction of iNKT cell anergy after αGalCer treatment, the secretion of both Th1 and Th2 cytokines by iNKT cells and immune suppression in the TME. There are several limitations and obstacles to the clinical translation of iNKT cell therapy into humans, and many strategies have been proposed to overcome these limitations (which have been reviewed extensively elsewhere) (9, 11, 90, 91, 93). These strategies include alternative vectors for the delivery of αGalCer (83, 94), the generation of more potent αGalCer analogues and other iNKT cell agonists (95), the generation of induced pluripotent stem cell-derived iNKT cells and improvements to iNKT-CAR based platforms (90, 96).

A further potential strategy proposed here is the selection and utilisation of specific human iNKT cell subsets to achieve improved outcomes in iNKT-based cancer immunotherapy. This proposal is based on studies revealing that iNKT cells exist as subpopulations with a previously unrecognised diversity in function (19, 25, 38, 97). iNKT cells are not a single, uniform class of T cells as they exhibit heterogeneity in both phenotype and function. In mice, iNKT cells seemingly exhibit at least three distinct thymic populations based on the expression of unique sets of transcription factors, namely iNKT1 (T-bet^+^), iNKT2 (GATA-3^+^) and iNKT17 (RORγt^+^) (98). While iNKT1 produce IFN-γ and also some IL-4, iNKT2 produce IL-4 and iNKT17 produce IL-17. BALB/c mice have large proportions of iNKT2/iNKT17 cells but reduced proportions of iNKT1 cells. C57BL/6 mice were highly enriched for iNKT1 but no other subsets, and NOD mice have equal proportions of all three subsets, revealing inter-strain variability (98). Further described subsets are iNKT_FH_ or follicular helper iNKT that provide cognate help for B cells (99, 100), and iNKT10 that play important roles in maintaining adipose tissue homeostasis (101).

Although most mouse iNKT cells express the canonical Vα14-Jα18 TCR α-chain, they can use different Vβ chains. Combinations of Vβ-, Jβ-, and CDR3β-encoded residues will ultimately determine the type of ligands that iNKT cells recognise (102, 103). Basal activation, proliferation, TCRβ repertoire and antigen specificity are seemingly modulated by their anatomical location (104). Thus, the so-called invariant NKT cell population expresses a variable TCRVβ repertoire that differs in antigen recognition in individual tissues. Importantly, anatomical differences also apply to human iNKT cells. For example, Jimeno et al. found increased frequencies of atypical iNKT cells (Vα24- or Vβ11-) in tonsils *vs*. blood, while the frequency of CD4^+^ and CD69^+^ iNKT cells was also different in those anatomical locations (104). The diverse iNKT subpopulations thus occupy and utilise unique anatomical and physiological niches to perform their diverse biological functions, likely to be context and tissue specific.

## Human iNKT cell subsets

**Figure 2:**
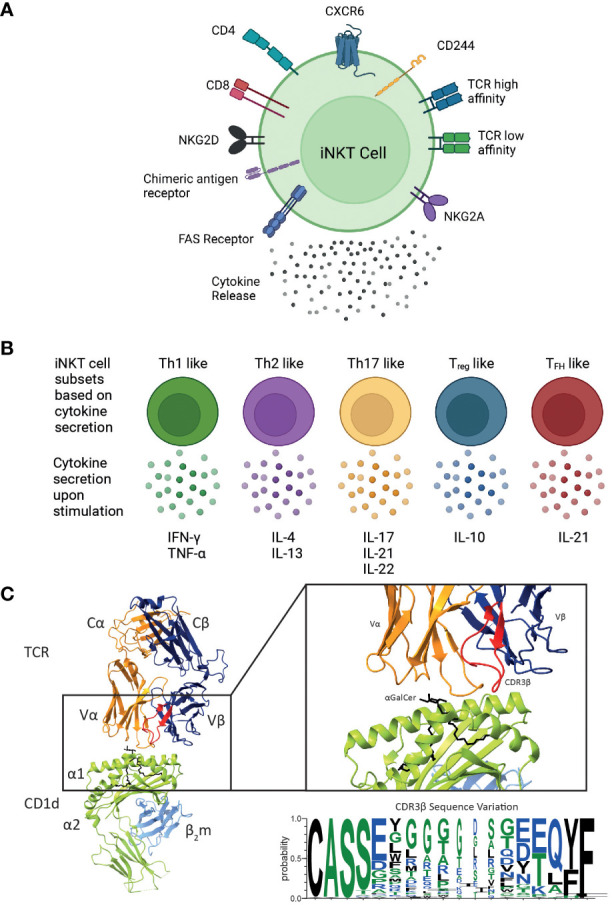
Overview of the different functional human iNKT cell subsets. CD1d-restricted human iNKT cells can be divided into distinct subsets based on their **(A)** cell surface receptor expression, T cell receptor (TCR) repertoire and cytokine profile. Human iNKT cells can express the glycoproteins CD4 and CD8, but the majority of human iNKT cells are either CD4^+^ or CD4^-^CD8^-^ (double negative), leading to four distinct subsets. They also express a variety of chemokine receptors and other cell surface molecules including NK receptors. Additionally, chimeric antigen receptors can be engineered on iNKT cells to target them to specific malignancies. Expression of various surface molecules has been found to confer enhanced cytotoxicity. **(B)** Upon activation, iNKT cells secrete a unique pattern of cytokines, indicated for each subtype. **(C)** iNKT cells are defined by the expression of their invariant TCR αβ, however variation within the CDR3β sequence (as shown in the sequence logo) confers differences in the affinity of the iNKT cell TCR CD1d-lipid interaction. These differences in phenotype, TCR affinity and cytokine profile are all likely to impact their cytotoxic responses *in vivo*. Cartoons created with BioRender.com. Molecular graphics and analyses performed with UCSF ChimeraX; PDB code 4EN3. Sequence logo generated using WebLogo 3 using the CDR3β sequences (97, 105, 106).

Mature human iNKT cells can be categorised according to numerous characteristics (**Figure 2**). Broadly, human iNKT cells are characterised by CD4 expression, as they exist as CD4^+^, and CD4^-^ subsets (25). CD4^-^ iNKTs are DN or CD8^+^with a very small subset of DP iNKT cells also identified (26, 107). The relative frequencies vary substantially between individuals: on average, CD4^+^ and DN cells are the most frequent subsets, and CD8^+^ iNKT cells have a low frequency (26, 108). There is evidence suggesting that DN human iNKT cells are different from their CD4^+^ iNKT cell counterparts (25, 107). DN iNKT cells are seemingly similar to mouse iNKT1 cells, exhibiting an increased IFN-γ secretion and cytotoxic function when activated (25, 107).

Although iNKT cell subsets share the expression profile for several chemokine receptors, they may also differ with respect to their chemokine receptor expression (**Figure 2A**). CD4^+^ iNKT cells predominantly express CCR4, while CD8 and DN iNKT cell subsets mainly express CCR1, CCR6 and CXCR6 (108). Based on these observations, a differential tissue distribution can be assumed; for example, CXCR6 plays a role in the homeostatic distribution of iNKT cells to the liver and the lung (109, 110). Human CD4^+^ iNKT cells are broadly associated with Th0-type immune responses and are the exclusive producers of IL-4 and IL-13 upon primary stimulation; whereas, DN iNKT cells have a strict Th1 profile and prominently express several NK lineage receptors (107). Additionally, it has been shown that CD244^+^ CXCR6^+^ iNKT cells, which are present in mice and humans, have enhanced cytotoxic properties producing more IFN-γ compared to CD244^-^ CXCR6^-^ iNKT cells (38). Intriguingly, this CD244^+^ CXCR6^+^ iNKT cell subset is CD4^-^, potentially explaining the enhanced cytotoxic function of the CD4^-^ iNKT cell subset (38).

In a B cell lymphoma model, Tian and colleagues demonstrated that CD19-specific CAR-iNKT cells expressing CD62L mediated tumour regression (111). CD62L is involved in homing of naïve and central memory T cells to secondary lymphoid organs. The Metelitsa group also showed that these CD62L^+^ iNKT cells have prolonged persistence and anti-tumour activity *in vivo* (111). Subsequently, IL-21 was demonstrated to preserve the crucial central memory-like iNKT subset and enhance anti-tumour effector functionality. Following antigenic stimulation with αGalCer, CD62L^+^ iNKT cells both expressed IL-21R and secreted IL-21, each at significantly higher levels than CD62L^-^ cells (111). Although IL-21 alone failed to expand stimulated iNKT cells, combined IL-2/IL-21 treatment produced more iNKT cells and increased the frequency of CD62L^+^ cells versus IL-2 alone. Gene expression analysis of CD62L^+^ and CD62L^-^ cells revealed that treatment with a combination of IL-2 and IL-21 (but not IL-2 alone) selectively downregulated the proapoptotic protein BIM in CD62L^+^ iNKT cells, thus protecting them from activation-induced cell death (111, 112). While these studies have been conducted in mice, evidence suggests that human iNKT cells can also express CD62L (113). It would be interesting therefore to evaluate the anti-tumour activity and persistence of human CD62L^+^ iNKT cells in future studies.

Based on their cytokine secretion pattern, human iNKT cells have also been classified into Th-cell subsets (**Figure 2B**). Th1-like iNKT cells have been identified in healthy individuals producing Th1-associated cytokines such as IFN-γ and TNF-α upon stimulation (25, 107, 114). These Th1-like iNKT cells are predominantly DN cells and express more NKG2D compared to CD4^+^ iNKT cells. In contrast, Th2-like iNKT cell subsets with regulatory properties tend to be CD4^+^ and they secrete IL-4, IL-13, and IFN-γ upon activation (25, 107). Human Th17-like iNKT cells secrete the proinflammatory cytokines IL-17, IL-21 and IL-22 when activated (115). Finally, FOXP3-expressing Treg-like iNKT cells that secrete the immunosuppressive cytokine IL-10, as well as T_FH_-like iNKT cells that secrete IL-21 upon activation have also been described (115, 116). However, iNKT cells exhibit plasticity in terms of their cytokine production and therefore the above-described definitions are not absolute.

Diversity within human and mouse iNKT cells is further observed based on the TCRβ sequence of the iNKT cell TCR. In mice, the iNKT repertoire displays clonal heterogeneity regarding lipid recognition, such as the αGalCer analogue OCH, which can be explained by the differential Vβ family usage in mouse iNKT cell TCRs (22, 117, 118). In contrast, the human iNKT cell TCR is composed of the invariant Vα24 TCRα chain and the semi-variant Vβ11 TCRβ chain, whereby the CDR3β region is the only truly adaptive element (**Figure 2C**). Thus, human iNKT cells in healthy adults express TCRs with widely variable affinities for CD1d, which are directly dependent on structural differences within the CDR3β loop of the iNKT cell TCR (97). Clonal variations resulted in up to a 40-fold difference in TCR affinity to CD1d and could be directly correlated to iNKT cell function (97). iNKT clones bearing high affinity iNKT cell TCRs proliferated more avidly and produced a greater diversity of cytokines in response to CD1d expressing APCs compared to clones expressing a low affinity iNKT cell TCR (97). iNKT cell TCR affinities are seemingly skewed in rheumatoid arthritis (119) and type 1 diabetes (120) which may also contribute to disease pathology in the context of human cancer. A recent study identified three CDR3β amino acid sequence motifs that were seemingly associated with strong autoreactivity: a VD region with two or more acidic amino acids; usage of the Jβ2-5 allele; and a 13 amino acid long CDR3β motif (105). Acidic amino acid composition, J usage, and the CDR3β region amino acid length individually affected the affinities of conventional TCRs (121–123). Additionally, the Hirano group revealed that the presence of a naturally encoded arginine (Arg) or a serine (Ser) in the third position of the CDR3β loop, can effectively modulate antigen recognition by the iNKT TCR (124). In agreement with previous studies, Ser to Arg substitutions influence the structure of the CDR3α loop thus effecting the iNKT cell TCR affinity (124). Furthermore, such differences in iNKT cell TCR affinities also influence iNKT cell function (124).

## Outlook

Studies in mice have revealed that iNKT cells can be exploited as a powerful platform for cancer treatment (4, 5). However, there are limitations to such studies which makes it difficult to reconcile data for an effective clinical translation of iNKT cell immunotherapy from mice to humans. Laboratory mice are inbred strains that lack genetic heterogeneity (125), and therefore conclusions from pre-clinical models might be exaggerated as it does not take into account the wide genetic variability of the human population. This is complicated by the fact that there are far more mouse studies conducted on iNKT cells and therefore, more studies investigating human iNKT cells are urgently needed. Although there are similarities between iNKT cells derived from mice and humans, there are also some key differences that should be taken into consideration when designing studies for clinical translation into humans. Mice have far more iNKT cells than humans and many studies have shown that targeting iNKT cells in mice induce powerful Th1 mediated anti-tumour immunity (4, 5). However, it is well known that iNKT cells are less frequent in healthy humans (126). Furthermore, cancer patients, particularly those with late stage disease, have reduced iNKT cell numbers and those that are present are often defective in their cytokine secretion, suggesting that cancer patients exhibit a profoundly immunocompromised iNKT cell repertoire (39, 49, 127, 128). It is likely that targeting an already exhausted and unresponsive iNKT cell repertoire in these patients may not lead to potent anti-tumour immune responses, potentially explaining the disappointing results in human iNKT cell-based clinical trials. Therefore, a better understanding of the human iNKT cell repertoire in healthy individuals and in cancer patients is needed to fully characterise human iNKT cell heterogeneity and its contribution in health and disease.

In addition, future iNKT cell targeting therapies should consider exploiting the specific iNKT cell subpopulations with respect to their diverse co-receptor expression phenotypes and TCR affinities. We propose that a better understanding of the mechanisms driving anti-tumour functions of diverse human iNKT cell subpopulations will achieve significant advances in their exploitation in cancer therapies which may lead to better clinical efficacy. Emerging multimodal approaches such as single cell RNA and TCR sequencing will be needed to determine the relevance of specific iNKT cell subsets and clonotypes, which will require better *in vivo* models that can accurately reflect human disease (129, 130). The combination of TCR sequencing data with the transcriptomic information of single antigen specific T cells has had a significant impact on understanding the heterogeneity of T cell populations (131–133). T cells that express the same TCR αβ undergo clonal expansions and can give rise to progeny with diverse functional phenotypes. Sequencing of the TCR repertoire can highlight clonal diversity and the dynamics of antigen specific responses associated with the anti-tumour response (134, 135). On the other hand, the transcriptomic information can reveal distinct functional phenotypes associated with better disease outcomes. Therefore, coupling these datasets together can reveal distinct T cell populations that are most relevant to disease states (134, 135). Indeed, the application of these technologies have advanced our knowledge of CD8^+^ and CD4^+^ T cell heterogeneity in response to Yellow Fever Virus vaccination in humans (132), peanut allergy (136), and in colorectal cancer (133). Advances in sequencing technologies in recent years allows for T cell clones to be tracked and monitored to assess their behaviour during infection or during anti-tumour responses which enables the tracing of the progeny of individual T cells back to their ancestors (131, 137–139). Fate mapping and lineage tracing can therefore be applied to iNKT cell populations to better understand iNKT cell population dynamics, clonal heterogeneity with regards to CDR3β diversity to highlight subpopulations of iNKT cells that are disease relevant. Furthermore, T cell barcoding (131) which allows linage tracing, can be applied to iNKT cell populations to assess inter-clonal differences and to monitor the contribution of individual clonal populations to effector functions during anti-tumour responses. Fate mapping and lineage tracing of iNKT cells may uncover inter-clonal diversity and could provide data on clonal contribution to the anti-tumour immune response. The application of these new technologies to human iNKT cells will provide high dimensional data that will allow a better understanding of human iNKT cell subpopulations in disease states, thus furthering our understanding of the diversity and complexity of the iNKT cell response in humans. Furthermore, it is imperative that such studies should be conducted in reliable preclinical humanised mouse models of cancer that display the human CD1d antigen presentation system, complemented by human iNKT cells (140, 141). Investigating the anti-tumour efficacy of diverse human iNKT cell subpopulations in these models and through utilising human cancer cells will likely provide a deeper understanding of human iNKT cell subsets in preclinical studies. In summary, exploiting human iNKT cells complement current cancer immunotherapies and as such knowledge of human iNKT cell subpopulations in the context of tumour immunology is urgently required to help improve the translational efficacy of future iNKT cell based immunotherapies.

## Author contributions

AL, DB, and SM developed the concept and wrote the manuscript. AL, generated all the figures and tables. All authors contributed to the article and approved the submitted version.

## References

[1] Robert C. A decade of immune-checkpoint inhibitors in cancer therapy. Nat Commun (2020) 11(1):3801. doi: 10.1038/s41467-020-17670-y 32732879 PMC7393098

[2] Jiang Y Zhao X Fu J Wang H. Progress and challenges in precise treatment of tumors with PD-1/PD-L1 blockade. Front Immunol (2020) 11:339. doi: 10.3389/fimmu.2020.00339 32226426 PMC7080697

[3] Roy R Singh SK Misra S. Advancements in cancer immunotherapies. Vaccines (Basel) (2022) 11(1):59. doi: 10.3390/vaccines11010059 36679904 PMC9861770

[4] Crowe NY Smyth MJ Godfrey DI. A critical role for natural killer T cells in immunosurveillance of methylcholanthrene-induced sarcomas. J Exp Med (2002) 196(1):119–27. doi: 10.1084/jem.20020092 PMC2194015 12093876

[5] Cui J Shin T Kawano T Sato H Kondo E Toura I. Requirement for Valpha14 NKT cells in IL-12-mediated rejection of tumors. Science (1997) 278(5343):1623–6. doi: 10.1126/science.278.5343.1623 9374462

[6] Rotolo A Caputo VS Holubova M Baxan N Dubois O Chaudhry MS. Enhanced anti-lymphoma activity of CAR19-iNKT cells underpinned by dual CD19 and CD1d targeting. Cancer Cell (2018) 34(4):596–610 e511. doi: 10.1016/j.ccell.2018.08.017 30300581 PMC6179961

[7] Rubio MT Bouillie M Bouazza N Coman T Trebeden-Negre H Gomez A. Pre-transplant donor CD4(-) invariant NKT cell expansion capacity predicts the occurrence of acute graft-versus-host disease. Leukemia (2017) 31(4):903–12. doi: 10.1038/leu.2016.281 27740636

[8] Chaidos A Patterson S Szydlo R Chaudhry MS Dazzi F Kanfer E. Graft invariant natural killer T-cell dose predicts risk of acute graft-versus-host disease in allogeneic hematopoietic stem cell transplantation. Blood (2012) 119(21):5030–6. doi: 10.1182/blood-2011-11-389304 PMC6143158 22371885

[9] Painter GF Burn OK Hermans IF. Using agonists for iNKT cells in cancer therapy. Mol Immunol (2021) 130:1–6. doi: 10.1016/j.molimm.2020.12.010 33340930

[10] Wolf BJ Choi JE Exley MA. Novel approaches to exploiting invariant NKT cells in cancer immunotherapy. Front Immunol (2018) 9:384. doi: 10.3389/fimmu.2018.00384 29559971 PMC5845557

[11] Nair S Dhodapkar MV. Natural killer T cells in cancer immunotherapy. Front Immunol (2017) 8:1178. doi: 10.3389/fimmu.2017.01178 29018445 PMC5614937

[12] Brennan PJ Brigl M Brenner MB. Invariant natural killer T cells: an innate activation scheme linked to diverse effector functions. Nat Rev Immunol (2013) 13(2):101–17. doi: 10.1038/nri3369 23334244

[13] Kawano T Cui J Koezuka Y Toura I Kaneko Y Motoki K. CD1d-restricted and TCR-mediated activation of valpha14 NKT cells by glycosylceramides. Science (1997) 278(5343):1626–9. doi: 10.1126/science.278.5343.1626 9374463

[14] Borg NA Wun KS Kjer-Nielsen L Wilce MC Pellicci DG Koh R. CD1d-lipid-antigen recognition by the semi-invariant NKT T-cell receptor. Nature (2007) 448(7149):44–9. doi: 10.1038/nature05907 17581592

[15] Salio M Silk JD Jones EY Cerundolo V. Biology of CD1- and MR1-restricted T cells. Annu Rev Immunol (2014) 32:323–66. doi: 10.1146/annurev-immunol-032713-120243 24499274

[16] Bae EA Seo H Kim IK Jeon I Kang CY. Roles of NKT cells in cancer immunotherapy. Arch Pharm Res (2019) 42(7):543–8. doi: 10.1007/s12272-019-01139-8 30859410

[17] Crowe NY Coquet JM Berzins SP Kyparissoudis K Keating R Pellicci DG. Differential antitumor immunity mediated by NKT cell subsets *in vivo* . J Exp Med (2005) 202(9):1279–88. doi: 10.1084/jem.20050953 PMC1459911 16275765

[18] Hung J-T Huang J-R Yu AL. Tailored design of NKT-stimulatory glycolipids for polarization of immune responses. J Biomed Sci (2017) 24(1):1–10. doi: 10.1186/s12929-017-0325-0 28335781 PMC5364570

[19] Kuylenstierna C Bjorkstrom NK Andersson SK Sahlstrom P Bosnjak L Paquin-Proulx D. NKG2D performs two functions in invariant NKT cells: direct TCR-independent activation of NK-like cytolysis and co-stimulation of activation by CD1d. Eur J Immunol (2011) 41(7):1913–23. doi: 10.1002/eji.200940278 PMC3523190 21590763

[20] Smyth MJ Crowe NY Pellicci DG Kyparissoudis K Kelly JM Takeda K. Sequential production of interferon-gamma by NK1.1(+) T cells and natural killer cells is essential for the antimetastatic effect of alpha-galactosylceramide. Blood (2002) 99(4):1259–66. doi: 10.1182/blood.v99.4.1259 11830474

[21] Shissler SC Webb TJ. The ins and outs of type I iNKT cell development. Mol Immunol (2019) 105:116–30. doi: 10.1016/j.molimm.2018.09.023 PMC6331266 30502719

[22] Pellicci DG Patel O Kjer-Nielsen L Pang SS Sullivan LC Kyparissoudis K. Differential recognition of CD1d-alpha-galactosyl ceramide by the V beta 8.2 and V beta 7 semi-invariant NKT T cell receptors. Immunity (2009) 31(1):47–59. doi: 10.1016/j.immuni.2009.04.018 19592275 PMC2765864

[23] Hogquist K Georgiev H. Recent advances in iNKT cell development. F1000Res (2020) 9. doi: 10.12688/f1000research.21378.1 PMC7043113 32148771

[24] Exley MA Wilson SB Balk SP. Isolation and functional use of human NKT cells. Curr Protoc Immunol (2017) 119:14 11 11–14 11 20. doi: 10.1002/cpim.33 29091262

[25] Gumperz JE Miyake S Yamamura T Brenner MB. Functionally distinct subsets of CD1d-restricted natural killer T cells revealed by CD1d tetramer staining. J Exp Med (2002) 195(5):625–36. doi: 10.1084/jem.20011786 PMC2193772 11877485

[26] Montoya CJ Pollard D Martinson J Kumari K Wasserfall C Mulder CB. Characterization of human invariant natural killer T subsets in health and disease using a novel invariant natural killer T cell-clonotypic monoclonal antibody, 6B11. Immunology (2007) 122(1):1–14. doi: 10.1111/j.1365-2567.2007.02647.x 17662044 PMC2265989

[27] Crosby CM Kronenberg M. Tissue-specific functions of invariant natural killer T cells. Nat Rev Immunol (2018) 18(9):559–74. doi: 10.1038/s41577-018-0034-2 PMC6343475 29967365

[28] Lantz O Bendelac A. An invariant T cell receptor alpha chain is used by a unique subset of major histocompatibility complex class I-specific CD4+ and CD4-8- T cells in mice and humans. J Exp Med (1994) 180(3):1097–106. doi: 10.1084/jem.180.3.1097 PMC2191643 7520467

[29] Stetson DB Mohrs M Reinhardt RL Baron JL Wang ZE Gapin L. Constitutive cytokine mRNAs mark natural killer (NK) and NK T cells poised for rapid effector function. J Exp Med (2003) 198(7):1069–76. doi: 10.1084/jem.20030630 PMC2194220 14530376

[30] Brigl M Bry L Kent SC Gumperz JE Brenner MB. Mechanism of CD1d-restricted natural killer T cell activation during microbial infection. Nat Immunol (2003) 4(12):1230–7. doi: 10.1038/ni1002 14578883

[31] Matsuda JL Mallevaey T Scott-Browne J Gapin L. CD1d-restricted iNKT cells, the ‘Swiss-army knife’ of the immune system. Curr Opin Immunol (2008) 20(3):358–68. doi: 10.1016/j.coi.2008.03.018 PMC2546701 18501573

[32] Crowe NY Uldrich AP Kyparissoudis K Hammond KJ Hayakawa Y Sidobre S. Glycolipid antigen drives rapid expansion and sustained cytokine production by NK T cells. J Immunol (2003) 171(8):4020–7. doi: 10.4049/jimmunol.171.8.4020 14530322

[33] Harada M Seino K Wakao H Sakata S Ishizuka Y Ito T. Down-regulation of the invariant Valpha14 antigen receptor in NKT cells upon activation. Int Immunol (2004) 16(2):241–7. doi: 10.1093/intimm/dxh023 14734609

[34] Metelitsa LS. Anti-tumor potential of type-I NKT cells against CD1d-positive and CD1d-negative tumors in humans. Clin Immunol (2011) 140(2):119–29. doi: 10.1016/j.clim.2010.10.005 PMC3444285 21095162

[35] Reilly EC Wands JR Brossay L. Cytokine dependent and independent iNKT cell activation. Cytokine (2010) 51(3):227–31. doi: 10.1016/j.cyto.2010.04.016 PMC2914806 20554220

[36] Liu TY Uemura Y Suzuki M Narita Y Hirata S Ohyama H. Distinct subsets of human invariant NKT cells differentially regulate T helper responses *via* dendritic cells. Eur J Immunol (2008) 38(4):1012–23. doi: 10.1002/eji.200737838 18350544

[37] Cerundolo V Silk JD Masri SH Salio M. Harnessing invariant NKT cells in vaccination strategies. Nat Rev Immunol (2009) 9(1):28–38. doi: 10.1038/nri2451 19079136

[38] Cui G Shimba A Jin J Ogawa T Muramoto Y Miyachi H. A circulating subset of iNKT cells mediates antitumor and antiviral immunity. Sci Immunol (2022) 7(76):eabj8760. doi: 10.1126/sciimmunol.abj8760 36269840

[39] Tahir SM Cheng O Shaulov A Koezuka Y Bubley GJ Wilson SB. Loss of IFN-gamma production by invariant NK T cells in advanced cancer. J Immunol (2001) 167(7):4046–50. doi: 10.4049/jimmunol.167.7.4046 11564825

[40] Hara A Koyama-Nasu R Takami M Toyoda T Aoki T Ihara F. CD1d expression in glioblastoma is a promising target for NKT cell-based cancer immunotherapy. Cancer Immunol Immunother (2020) 70(4):1239–54. doi: 10.1007/s00262-020-02742-1 PMC8053161 33128583

[41] Waldowska M Bojarska-Junak A Rolinski J. A brief review of clinical trials involving manipulation of invariant NKT cells as a promising approach in future cancer therapies. Cent Eur J Immunol (2017) 42(2):181–95. doi: 10.5114/ceji.2017.69361 PMC5573892 28860937

[42] Haraguchi K Takahashi T Nakahara F Matsumoto A Kurokawa M Ogawa S. CD1d expression level in tumor cells is an important determinant for anti-tumor immunity by natural killer T cells. Leuk Lymphoma (2006) 47(10):2218–23. doi: 10.1080/10428190600682688 17071498

[43] Hix LM Shi YH Brutkiewicz RR Stein PL Wang CR Zhang M. CD1d-expressing breast cancer cells modulate NKT cell-mediated antitumor immunity in a murine model of breast cancer metastasis. PloS One (2011) 6(6):e20702. doi: 10.1371/journal.pone.0020702 21695190 PMC3113806

[44] Fallarini S Paoletti T Orsi Battaglini N Lombardi G. Invariant NKT cells increase drug-induced osteosarcoma cell death. Br J Pharmacol (2012) 167(7):1533–49. doi: 10.1111/j.1476-5381.2012.02108.x PMC3514765 22817659

[45] Nicol A Nieda M Koezuka Y Porcelli S Suzuki K Tadokoro K. Human invariant valpha24+ natural killer T cells activated by alpha-galactosylceramide (KRN7000) have cytotoxic anti-tumour activity through mechanisms distinct from T cells and natural killer cells. Immunology (2000) 99(2):229–34. doi: 10.1046/j.1365-2567.2000.00952.x PMC2327139 10692041

[46] Spanoudakis E Hu M Naresh K Terpos E Melo V Reid A. Regulation of multiple myeloma survival and progression by CD1d. Blood (2009) 113(11):2498–507. doi: 10.1182/blood-2008-06-161281 19056691

[47] Díaz-Basabe A Strati F Facciotti F. License to kill: when iNKT cells are granted the use of lethal cytotoxicity. Int J Mol Sci (2020) 21(11):3909. doi: 10.3390/ijms21113909 32486268 PMC7312231

[48] Anthony DA Andrews DM Watt SV Trapani JA Smyth MJ. Functional dissection of the granzyme family: cell death and inflammation. Immunol Rev (2010) 235(1):73–92. doi: 10.1111/j.0105-2896.2010.00907.x 20536556

[49] Konishi J Yamazaki K Yokouchi H Shinagawa N Iwabuchi K Nishimura M. The characteristics of human NKT cells in lung cancer–CD1d independent cytotoxicity against lung cancer cells by NKT cells and decreased human NKT cell response in lung cancer patients. Hum Immunol (2004) 65(11):1377–88. doi: 10.1016/j.humimm.2004.09.003 15556688

[50] Voskoboinik I Whisstock JC Trapani JA. Perforin and granzymes: function, dysfunction and human pathology. Nat Rev Immunol (2015) 15(6):388–400. doi: 10.1038/nri3839 25998963

[51] Thiery J Keefe D Boulant S Boucrot E Walch M Martinvalet D. Perforin pores in the endosomal membrane trigger the release of endocytosed granzyme b into the cytosol of target cells. Nat Immunol (2011) 12(8):770–7. doi: 10.1038/ni.2050 PMC3140544 21685908

[52] Sparrow E Bodman-Smith MD. Granulysin: the attractive side of a natural born killer. Immunol Lett (2020) 217:126–32. doi: 10.1016/j.imlet.2019.11.005 31726187

[53] Peña SV Krensky AM. Granulysin, a new human cytolytic granule-associated protein with possible involvement in cell-mediated cytotoxicity. Semin Immunol (1997) 9(2):117–25. doi: 10.1006/smim.1997.0061 9194222

[54] Kishi A Takamori Y Ogawa K Takano S Tomita S Tanigawa M. Differential expression of granulysin and perforin by NK cells in cancer patients and correlation of impaired granulysin expression with progression of cancer. Cancer Immunol Immunother (2002) 50(11):604–14. doi: 10.1007/s002620100228 PMC11032915 11807624

[55] Saigusa S Ichikura T Tsujimoto H Sugasawa H Majima T Kawarabayashi N. Serum granulysin level as a novel prognostic marker in patients with gastric carcinoma. J Gastroenterol Hepatol (2007) 22(8):1322–7. doi: 10.1111/j.1440-1746.2006.04796.x 17688669

[56] Kaspar AA Okada S Kumar J Poulain FR Drouvalakis KA Kelekar A. A distinct pathway of cell-mediated apoptosis initiated by granulysin. J Immunol (2001) 167(1):350. doi: 10.4049/jimmunol.167.1.350 11418670

[57] Parekh VV Lalani S Van Kaer L. The in vivo response of invariant natural killer T cells to glycolipid antigens. Int Rev Immunol (2007) 26(1-2):31–48. doi: 10.1080/08830180601070179 17454263

[58] Fujii S Shimizu K Hemmi H Steinman RM. Innate Valpha14(+) natural killer T cells mature dendritic cells, leading to strong adaptive immunity. Immunol Rev (2007) 220:183–98. doi: 10.1111/j.1600-065X.2007.00561.x 17979847

[59] Smyth MJ Swann J Kelly JM Cretney E Yokoyama WM Diefenbach A. NKG2D recognition and perforin effector function mediate effective cytokine immunotherapy of cancer. J Exp Med (2004) 200(10):1325–35. doi: 10.1084/jem.20041522 PMC2211920 15545356

[60] Serafini P Borrello I Bronte V. Myeloid suppressor cells in cancer: recruitment, phenotype, properties, and mechanisms of immune suppression. Semin Cancer Biol (2006) 16(1):53–65. doi: 10.1016/j.semcancer.2005.07.005 16168663

[61] Allavena P Sica A Solinas G Porta C Mantovani A. The inflammatory micro-environment in tumor progression: the role of tumor-associated macrophages. Crit Rev Oncol Hematol (2008) 66(1):1–9. doi: 10.1016/j.critrevonc.2007.07.004 17913510

[62] Whiteside TL. The tumor microenvironment and its role in promoting tumor growth. Oncogene (2008) 27(45):5904–12. doi: 10.1038/onc.2008.271 PMC3689267 18836471

[63] Coussens LM Zitvogel L Palucka AK. Neutralizing tumor-promoting chronic inflammation: a magic bullet? Science (2013) 339(6117):286–91. doi: 10.1126/science.1232227 PMC3591506 23329041

[64] Qian BZ Pollard JW. Macrophage diversity enhances tumor progression and metastasis. Cell (2010) 141(1):39–51. doi: 10.1016/j.cell.2010.03.014 20371344 PMC4994190

[65] Song L Asgharzadeh S Salo J Engell K Wu HW Sposto R. Valpha24-invariant NKT cells mediate antitumor activity *via* killing of tumor-associated macrophages. J Clin Invest (2009) 119(6):1524–36. doi: 10.1172/JCI37869 PMC2689106 19411762

[66] Gabrilovich DI Nagaraj S. Myeloid-derived suppressor cells as regulators of the immune system. Nat Rev Immunol (2009) 9(3):162–74. doi: 10.1038/nri2506 PMC2828349 19197294

[67] De Santo C Salio M Masri SH Lee LY Dong T Speak AO. Invariant NKT cells reduce the immunosuppressive activity of influenza a virus-induced myeloid-derived suppressor cells in mice and humans. J Clin Invest (2008) 118(12):4036–48. doi: 10.1172/JCI36264 PMC2582442 19033672

[68] Kawano T Cui J Koezuka Y Toura I Kaneko Y Sato H. Natural killer-like nonspecific tumor cell lysis mediated by specific ligand-activated Vα14 NKT cells. Proc Natl Acad Sci (1998) 95(10):5690. doi: 10.1073/pnas.95.10.5690 9576945 PMC20440

[69] Morita M Motoki K Akimoto K Natori T Sakai T Sawa E. Structure-activity relationship of alpha-galactosylceramides against B16-bearing mice. J Med Chem (1995) 38(12):2176–87. doi: 10.1021/jm00012a018 7783149

[70] Wu L Yun Z Tagawa T de la Maza L Wu MO Yu J. Activation of CD1d-restricted natural killer T cells can inhibit cancer cell proliferation during chemotherapy by promoting the immune responses in murine mesothelioma. Cancer Immunol Immunother (2014) 63(12):1285–96. doi: 10.1007/s00262-014-1597-9 PMC11029433 25183171

[71] Nakagawa R Motoki K Ueno H Iijima R Nakamura H Kobayashi E. Treatment of hepatic metastasis of the Colon26 adenocarcinoma with an α-galactosylceramide, KRN7000. Cancer Res (1998) 58(6):1202. 9515806

[72] Fuji N Ueda Y Fujiwara H Toh T Yoshimura T Yamagishi H. Antitumor effect of alpha-galactosylceramide (KRN7000) on spontaneous hepatic metastases requires endogenous interleukin 12 in the liver. Clin Cancer Res (2000) 6(8):3380–7. 10955826

[73] Toura I Kawano T Akutsu Y Nakayama T Ochiai T Taniguchi M. Cutting edge: inhibition of experimental tumor metastasis by dendritic cells pulsed with alpha-galactosylceramide. J Immunol (1999) 163(5):2387–91. doi: 10.4049/jimmunol.163.5.2387 10452972

[74] Shin T Nakayama T Akutsu Y Motohashi S Shibata Y Harada M. Inhibition of tumor metastasis by adoptive transfer of IL-12-activated Valpha14 NKT cells. Int J Cancer (2001) 91(4):523–8. doi: 10.1002/1097-0215(20010215)91:4<523::AID-IJC1087>3.0.CO;2-L 11251976

[75] Li YR Zhou Y Wilson M Kramer A Hon R Zhu Y. Tumor-localized administration of alpha-GalCer to recruit invariant natural killer T cells and enhance their antitumor activity against solid tumors. Int J Mol Sci (2022) 23(14):7547. doi: 10.3390/ijms23147547 35886891 PMC9317565

[76] Bedel R Matsuda JL Brigl M White J Kappler J Marrack P. Lower TCR repertoire diversity in Traj18-deficient mice. Nat Immunol (2012) 13(8):705–6. doi: 10.1038/ni.2347 PMC3748587 22814339

[77] Xie J Pan Y Tao H Wang P Chen Y Gao J. Deficiency of mucosal-associated invariant T cells in TCRJα18 germline knockout mice. Immunohorizons (2019) 3(6):203–7. doi: 10.4049/immunohorizons.1900035 PMC6829908 31356166

[78] Dashtsoodol N Shigeura T Ozawa R Harada M Kojo S Watanabe T. Generation of novel Traj18-deficient mice lacking Valpha14 natural killer T cells with an undisturbed T cell receptor alpha-chain repertoire. PloS One (2016) 11(4):e0153347. doi: 10.1371/journal.pone.0153347 27064277 PMC4827811

[79] Giaccone G Punt CJ Ando Y Ruijter R Nishi N Peters M. A phase I study of the natural killer T-cell ligand alpha-galactosylceramide (KRN7000) in patients with solid tumors. Clin Cancer Res (2002) 8(12):3702–9. 12473579

[80] Chang DH Osman K Connolly J Kukreja A Krasovsky J Pack M. Sustained expansion of NKT cells and antigen-specific T cells after injection of alpha-galactosyl-ceramide loaded mature dendritic cells in cancer patients. J Exp Med (2005) 201(9):1503–17. doi: 10.1084/jem.20042592 PMC1389847 15867097

[81] Ishikawa A Motohashi S Ishikawa E Fuchida H Higashino K Otsuji M. A phase I study of alpha-galactosylceramide (KRN7000)-pulsed dendritic cells in patients with advanced and recurrent non-small cell lung cancer. Clin Cancer Res (2005) 11(5):1910–7. doi: 10.1158/1078-0432.CCR-04-1453 15756017

[82] Okita K Motohashi S Shinnakasu R Nagato K Yamasaki K Sato Y. A set of genes associated with the interferon-gamma response of lung cancer patients undergoing alpha-galactosylceramide-pulsed dendritic cell therapy. Cancer Sci (2010) 101(11):2333–40. doi: 10.1111/j.1349-7006.2010.01696.x PMC11159413 20804502

[83] Nagato K Motohashi S Ishibashi F Okita K Yamasaki K Moriya Y. Accumulation of activated invariant natural killer T cells in the tumor microenvironment after alpha-galactosylceramide-pulsed antigen presenting cells. J Clin Immunol (2012) 32(5):1071–81. doi: 10.1007/s10875-012-9697-9 22534863

[84] Richter J Neparidze N Zhang L Nair S Monesmith T Sundaram R. Clinical regressions and broad immune activation following combination therapy targeting human NKT cells in myeloma. Blood (2013) 121(3):423–30. doi: 10.1182/blood-2012-06-435503 PMC3548165 23100308

[85] Toyoda T Kamata T Tanaka K Ihara F Takami M Suzuki H. Phase II study of alpha-galactosylceramide-pulsed antigen-presenting cells in patients with advanced or recurrent non-small cell lung cancer. J Immunother Cancer (2020) 8(1):e000316. doi: 10.1136/jitc-2019-000316 32188702 PMC7078938

[86] Cheng X Wang J Qiu C Jin Y Xia B Qin R. Feasibility of iNKT cell and PD-1+CD8+ T cell-based immunotherapy in patients with lung adenocarcinoma: preliminary results of a phase I/II clinical trial. Clin Immunol (2022) 238:108992. doi: 10.1016/j.clim.2022.108992 35367396

[87] Motohashi S Okamoto Y Yoshino I Nakayama T. Anti-tumor immune responses induced by iNKT cell-based immunotherapy for lung cancer and head and neck cancer. Clin Immunol (2011) 140(2):167–76. doi: 10.1016/j.clim.2011.01.009 21349771

[88] Exley MA Friedlander P Alatrakchi N Vriend L Yue S Sasada T. Adoptive transfer of invariant NKT cells as immunotherapy for advanced melanoma: a phase I clinical trial. Clin Cancer Res (2017) 23(14):3510–9. doi: 10.1158/1078-0432.CCR-16-0600 PMC5511564 28193627

[89] Heczey A Liu D Tian G Courtney AN Wei J Marinova E. Invariant NKT cells with chimeric antigen receptor provide a novel platform for safe and effective cancer immunotherapy. Blood (2014) 124(18):2824–33. doi: 10.1182/blood-2013-11-541235 PMC4215313 25049283

[90] Liu Y Wang G Chai D Dang Y Zheng J Li H. iNKT: a new avenue for CAR-based cancer immunotherapy. Transl Oncol (2022) 17:101342. doi: 10.1016/j.tranon.2022.101342 35063813 PMC8784340

[91] Nelson A Lukacs JD Johnston B. The current landscape of NKT cell immunotherapy and the hills ahead. Cancers (Basel) (2021) 13(20):5174. doi: 10.3390/cancers13205174 34680322 PMC8533824

[92] Molling JW Kölgen W van der Vliet HJ Boomsma MF Kruizenga H Smorenburg CH. Peripheral blood IFN-gamma-secreting Valpha24+Vbeta11+ NKT cell numbers are decreased in cancer patients independent of tumor type or tumor load. Int J Cancer (2005) 116(1):87–93. doi: 10.1002/ijc.20998 15756674

[93] Delfanti G Dellabona P Casorati G Fedeli M. Adoptive immunotherapy with engineered iNKT cells to target cancer cells and the suppressive microenvironment. Front Med (Lausanne) (2022) 9:897750. doi: 10.3389/fmed.2022.897750 35615083 PMC9125179

[94] Nicol AJ Tazbirkova A Nieda M. Comparison of clinical and immunological effects of intravenous and intradermal administration of alpha-galactosylceramide (KRN7000)-pulsed dendritic cells. Clin Cancer Res (2011) 17(15):5140–51. doi: 10.1158/1078-0432.CCR-10-3105 21653690

[95] Carreno LJ Saavedra-Avila NA Porcelli SA. Synthetic glycolipid activators of natural killer T cells as immunotherapeutic agents. Clin Transl Immunol (2016) 5(4):e69. doi: 10.1038/cti.2016.14 PMC4855264 27195112

[96] Kitayama S Zhang R Liu TY Ueda N Iriguchi S Yasui Y. Cellular adjuvant properties, direct cytotoxicity of re-differentiated Valpha24 invariant NKT-like cells from human induced pluripotent stem cells. Stem Cell Rep (2016) 6(2):213–27. doi: 10.1016/j.stemcr.2016.01.005 PMC4750166 26862702

[97] Matulis G Sanderson JP Lissin NM Asparuhova MB Bommineni GR Schumperli D. Innate-like control of human iNKT cell autoreactivity *via* the hypervariable CDR3beta loop. PloS Biol (2010) 8(6):e1000402. doi: 10.1371/journal.pbio.1000402 20585371 PMC2889927

[98] Lee YJ Holzapfel KL Zhu J Jameson SC Hogquist KA. Steady-state production of IL-4 modulates immunity in mouse strains and is determined by lineage diversity of iNKT cells. Nat Immunol (2013) 14(11):1146–54. doi: 10.1038/ni.2731 PMC3824254 24097110

[99] Chang PP Barral P Fitch J Pratama A Ma CS Kallies A. Identification of bcl-6-dependent follicular helper NKT cells that provide cognate help for b cell responses. Nat Immunol (2011) 13(1):35–43. doi: 10.1038/ni.2166 22120117

[100] King IL Fortier A Tighe M Dibble J Watts GF Veerapen N. Invariant natural killer T cells direct b cell responses to cognate lipid antigen in an IL-21-dependent manner. Nat Immunol (2011) 13(1):44–50. doi: 10.1038/ni.2172 22120118 PMC3833037

[101] Lynch L Michelet X Zhang S Brennan PJ Moseman A Lester C. Regulatory iNKT cells lack expression of the transcription factor PLZF and control the homeostasis of t(reg) cells and macrophages in adipose tissue. Nat Immunol (2015) 16(1):85–95. doi: 10.1038/ni.3047 25436972 PMC4343194

[102] Cameron G Pellicci DG Uldrich AP Besra GS Illarionov P Williams SJ. Antigen specificity of type I NKT cells is governed by TCR beta-chain diversity. J Immunol (2015) 195(10):4604–14. doi: 10.4049/jimmunol.1501222 26423148

[103] Mallevaey T Scott-Browne JP Matsuda JL Young MH Pellicci DG Patel O. T Cell receptor CDR2 beta and CDR3 beta loops collaborate functionally to shape the iNKT cell repertoire. Immunity (2009) 31(1):60–71. doi: 10.1016/j.immuni.2009.05.010 19592274 PMC2965025

[104] Jimeno R Lebrusant-Fernandez M Margreitter C Lucas B Veerapen N Kelly G. Tissue-specific shaping of the TCR repertoire and antigen specificity of iNKT cells. Elife (2019) 8:e51663. doi: 10.7554/eLife.51663 31841113 PMC6930077

[105] Chamoto K Guo T Imataki O Tanaka M Nakatsugawa M Ochi T. CDR3β sequence motifs regulate autoreactivity of human invariant NKT cell receptors. J Autoimmun (2016) 68:39–51. doi: 10.1016/j.jaut.2015.12.005 26748722 PMC4792736

[106] Rossjohn J Pellicci DG Patel O Gapin L Godfrey DI. Recognition of CD1d-restricted antigens by natural killer T cells. Nat Rev Immunol (2012) 12(12):845–57. doi: 10.1038/nri3328 PMC3740582 23154222

[107] Lee PT Benlagha K Teyton L Bendelac A. Distinct functional lineages of human V(alpha)24 natural killer T cells. J Exp Med (2002) 195(5):637–41. doi: 10.1084/jem.20011908 PMC2193771 11877486

[108] Kim CH Johnston B Butcher EC. Trafficking machinery of NKT cells: shared and differential chemokine receptor expression among V alpha 24(+)V beta 11(+) NKT cell subsets with distinct cytokine-producing capacity. Blood (2002) 100(1):11–6. doi: 10.1182/blood-2001-12-0196 12070001

[109] Germanov E Veinotte L Cullen R Chamberlain E Butcher EC Johnston B. Critical role for the chemokine receptor CXCR6 in homeostasis and activation of CD1d-restricted NKT cells. J Immunol (2008) 181(1):81–91. doi: 10.4049/jimmunol.181.1.81 18566372

[110] Shimaoka T Seino K Kume N Minami M Nishime C Suematsu M. Critical role for CXC chemokine ligand 16 (SR-PSOX) in Th1 response mediated by NKT cells. J Immunol (2007) 179(12):8172–9. doi: 10.4049/jimmunol.179.12.8172 18056360

[111] Tian G Courtney AN Jena B Heczey A Liu D Marinova E. CD62L+ NKT cells have prolonged persistence and antitumor activity *in vivo* . J Clin Invest (2016) 126(6):2341–55. doi: 10.1172/JCI83476 PMC4887157 27183388

[112] Ngai H Tian G Courtney AN Ravari SB Guo L Liu B. IL-21 selectively protects CD62L(+) NKT cells and enhances their effector functions for adoptive immunotherapy. J Immunol (2018) 201(7):2141–53. doi: 10.4049/jimmunol.1800429 PMC6143411 30111631

[113] Trujillo-Ocampo A Cho HW Clowers M Pareek S Ruiz-Vazquez W Lee SE. IL-7 during antigenic stimulation using allogeneic dendritic cells promotes expansion of CD45RA(-)CD62L(+)CD4(+) invariant NKT cells with Th-2 biased cytokine production profile. Front Immunol (2020) 11:567406. doi: 10.3389/fimmu.2020.567406 33329531 PMC7728799

[114] Krijgsman D Hokland M Kuppen PJK. The role of natural killer T cells in cancer-a phenotypical and functional approach. Front Immunol (2018) 9:367–7. doi: 10.3389/fimmu.2018.00367 PMC5835336 29535734

[115] Snyder-Cappione JE Tincati C Eccles-James IG Cappione AJ Ndhlovu LC Koth LL. A comprehensive ex vivo functional analysis of human NKT cells reveals production of MIP1-α and MIP1-β, a lack of IL-17, and a Th1-bias in males. PloS One (2010) 5(11):e15412. doi: 10.1371/journal.pone.0015412 21082024 PMC2972714

[116] Moreira-Teixeira L Resende M Devergne O Herbeuval JP Hermine O Schneider E. Rapamycin combined with TGF-β converts human invariant NKT cells into suppressive Foxp3+ regulatory cells. J Immunol (2012) 188(2):624–31. doi: 10.4049/jimmunol.1102281 22156591

[117] Schümann J Voyle RB Wei BY MacDonald HR. Cutting edge: influence of the TCR V beta domain on the avidity of CD1d:alpha-galactosylceramide binding by invariant V alpha 14 NKT cells. J Immunol (2003) 170(12):5815–9. doi: 10.4049/jimmunol.170.12.5815 12794105

[118] Stanic AK Shashidharamurthy R Bezbradica JS Matsuki N Yoshimura Y Miyake S. Another view of T cell antigen recognition: cooperative engagement of glycolipid antigens by Va14Ja18 natural T(iNKT) cell receptor [corrected]. J Immunol (2003) 171(9):4539–51. doi: 10.4049/jimmunol.171.9.4539 14568927

[119] Mansour S Tocheva AS Sanderson JP Goulston LM Platten H Serhal L. Structural and functional changes of the invariant NKT clonal repertoire in early rheumatoid arthritis. J Immunol (2015) 195(12):5582–91. doi: 10.4049/jimmunol.1501092 PMC4671310 26553073

[120] Tocheva AS Mansour S Holt TG Jones S Chancellor A Sanderson JP. The clonal invariant NKT cell repertoire in people with type 1 diabetes is characterized by a loss of clones expressing high-affinity TCRs. J Immunol (2017) 198(4):1452–9. doi: 10.4049/jimmunol.1600255 28062695

[121] Udyavar A Alli R Nguyen P Baker L Geiger TL. Subtle affinity-enhancing mutations in a myelin oligodendrocyte glycoprotein-specific TCR alter specificity and generate new self-reactivity. J Immunol (2009) 182(7):4439–47. doi: 10.4049/jimmunol.0804377 PMC2681418 19299745

[122] Yokosuka T Takase K Suzuki M Nakagawa Y Taki S Takahashi H. Predominant role of T cell receptor (TCR)-alpha chain in forming preimmune TCR repertoire revealed by clonal TCR reconstitution system. J Exp Med (2002) 195(8):991–1001. doi: 10.1084/jem.20010809 11956290 PMC2193687

[123] Reynolds C Chong D Raynsford E Quigley K Kelly D Llewellyn-Hughes J. Elongated TCR alpha chain CDR3 favors an altered CD4 cytokine profile. BMC Biol (2014) 12:32. doi: 10.1186/1741-7007-12-32 24886643 PMC4046507

[124] Chamoto K Guo T Scally SW Kagoya Y Anczurowski M Wang CH. Key residues at third CDR3beta position impact structure and antigen recognition of human invariant NK TCRs. J Immunol (2017) 198(3):1056–65. doi: 10.4049/jimmunol.1601556 PMC5262525 28003379

[125] Chebib J Jackson BC Lopez-Cortegano E Tautz D Keightley PD. Inbred lab mice are not isogenic: genetic variation within inbred strains used to infer the mutation rate per nucleotide site. Heredity (Edinb) (2021) 126(1):107–16. doi: 10.1038/s41437-020-00361-1 PMC7852876 32868871

[126] Bienemann K Iouannidou K Schoenberg K Krux F Reuther S Feyen O. iNKT cell frequency in peripheral blood of Caucasian children and adolescent: the absolute iNKT cell count is stable from birth to adulthood. Scand J Immunol (2011) 74(4):406–11. doi: 10.1111/j.1365-3083.2011.02591.x 21671972

[127] Kawano T Nakayama T Kamada N Kaneko Y Harada M Ogura N. Antitumor cytotoxicity mediated by ligand-activated human V alpha24 NKT cells. Cancer Res (1999) 59(20):5102–5. 10537282

[128] Yanagisawa K Exley MA Jiang X Ohkochi N Taniguchi M Seino K. Hyporesponsiveness to natural killer T-cell ligand alpha-galactosylceramide in cancer-bearing state mediated by CD11b+ gr-1+ cells producing nitric oxide. Cancer Res (2006) 66(23):11441–6. doi: 10.1158/0008-5472.CAN-06-0944 17145891

[129] Saavedra-Avila NA Dellabona P Casorati G Veerapen N Besra GS Howell AR. A humanized mouse model for *in vivo* evaluation of invariant natural killer T cell responses. Front Immunol (2022) 13:1011209. doi: 10.3389/fimmu.2022.1011209 36263021 PMC9574442

[130] Wen X Kim S Xiong R Li M Lawrenczyk A Huang X. A subset of CD8alphabeta+ invariant NKT cells in a humanized mouse model. J Immunol (2015) 195(4):1459–69. doi: 10.4049/jimmunol.1500574 PMC4530047 26157173

[131] Gerlach C van Heijst JW Swart E Sie D Armstrong N Kerkhoven RM. One naive T cell, multiple fates in CD8+ T cell differentiation. J Exp Med (2010) 207(6):1235–46. doi: 10.1084/jem.20091175 PMC2882844 20479114

[132] Mold JE Modolo L Hard J Zamboni M Larsson AJM Stenudd M. Divergent clonal differentiation trajectories establish CD8(+) memory T cell heterogeneity during acute viral infections in humans. Cell Rep (2021) 35(8):109174. doi: 10.1016/j.celrep.2021.109174 34038736

[133] Han A Glanville J Hansmann L Davis MM. Linking T-cell receptor sequence to functional phenotype at the single-cell level. Nat Biotechnol (2014) 32(7):684–92. doi: 10.1038/nbt.2938 PMC4337815 24952902

[134] Schumacher TN Gerlach C van Heijst JW. Mapping the life histories of T cells. Nat Rev Immunol (2010) 10(9):621–31. doi: 10.1038/nri2822 20689559

[135] Al Khabouri S Gerlach C. T Cell fate mapping and lineage tracing technologies probing clonal aspects underlying the generation of CD8 T cell subsets. Scand J Immunol (2020) 92(6):e12983. doi: 10.1111/sji.12983 33037653 PMC7757170

[136] Tu AA Gierahn TM Monian B Morgan DM Mehta NK Ruiter B. TCR sequencing paired with massively parallel 3’ RNA-seq reveals clonotypic T cell signatures. Nat Immunol (2019) 20(12):1692–9. doi: 10.1038/s41590-019-0544-5 PMC7528220 31745340

[137] van Heijst JW Gerlach C Swart E Sie D Nunes-Alves C Kerkhoven RM. Recruitment of antigen-specific CD8+ T cells in response to infection is markedly efficient. Science (2009) 325(5945):1265–9. doi: 10.1126/science.1175455 19729659

[138] Schepers K Swart E van Heijst JW Gerlach C Castrucci M Sie D. Dissecting T cell lineage relationships by cellular barcoding. J Exp Med (2008) 205(10):2309–18. doi: 10.1084/jem.20072462 PMC2556794 18809713

[139] Fennell KA Vassiliadis D Lam EYN Martelotto LG Balic JJ Hollizeck S. Non-genetic determinants of malignant clonal fitness at single-cell resolution. Nature (2022) 601(7891):125–31. doi: 10.1038/s41586-021-04206-7 34880496

[140] Chuprin J Buettner H Seedhom MO Greiner DL Keck JG Ishikawa F. Humanized mouse models for immuno-oncology research. Nat Rev Clin Oncol (2023) 20(3):192–206. doi: 10.1038/s41571-022-00721-2 PMC10593256 36635480

[141] Roghanian A Hu G Fraser C Singh M Foxall RB Meyer MJ. Cyclophosphamide enhances cancer antibody immunotherapy in the resistant bone marrow niche by modulating macrophage FcγR expression. Cancer Immunol Res (2019) 7(11):1876–90. doi: 10.1158/2326-6066.CIR-18-0835 PMC7780711 31451483

